# Whole-Genome Shotgun Sequence of *Salmonella bongori*, First Isolated in Northwestern Italy

**DOI:** 10.1128/genomeA.00560-17

**Published:** 2017-07-06

**Authors:** Angelo Romano, Alberto Bellio, Guerrino Macori, Paul D. Cotter, Daniela Manila Bianchi, Silvia Gallina, Lucia Decastelli

**Affiliations:** a Food Control and Production Hygiene Unit, Istituto Zooprofilattico Sperimentale del Piemonte, Liguria e Valle d'Aosta, Turin, Italy; b Teagasc Food Research Centre, Moorepark, Fermoy, Cork, Ireland; c APC Microbiome Institute, Cork, Ireland

## Abstract

This study describes the whole-genome shotgun sequence of *Salmonella bongori* 48:z35:–, originally isolated from a 1-year-old symptomatic patient in northwest Italy, a typically nonendemic area. The draft genome sequence contained 4.56 Mbp and the G+C content was 51.27%.

## GENOME ANNOUNCEMENT

*Salmonella bongori* 48:z35:– is considered endemic in Sicily, Italy but has not been reported as a clinical isolate responsible for human infection in mainland Italy (1). Phylogenetic analysis and comprehensive *Salmonella* evolution studies have suggested the genomic relationship between *S. bongori* and an ancestral *Salmonella* virulence gene set, which has been adapted by incorporating virulence determinants highly similar to those found in enteropathogenic *Escherichia coli* (2). Symptoms, in human cases, include nausea, fever, vomiting, abdominal pain, diarrhea, and acute enteritis (3).

Here, we report the whole-genome shotgun sequence of *S. bongori*, a fecal isolate from a 1-year old symptomatic patient in Piedmont in northwestern of Italy. The genomic content of the isolates was analyzed for potential genes that may be involved in virulence and antibiotic resistance. Bacterial genomic DNA was extracted using the DNeasy blood and tissue kit (Qiagen, USA), the genomic libraries were prepared using the Nextera XT library prep kit, and 250-bp paired-end sequencing was performed using the Illumina MiSeq platform (Illumina, San Diego, CA). Raw reads were preprocessed to remove adapter sequences and low-quality reads using Trimmomatic (v0.36) software (4). *De novo* assembly was performed using the SPAdes genome assembler (v3.9.1) (5). Assembly resulted in the generation of 85 contigs with a total size of 4.57 Mb, a G+C content of 51.3%, and an *N*_50_ of 179,074 bp. After assembly, gene prediction was performed using Prodigal (6). As a result, a total number of 4,242 genes were predicted. The draft genome sequence was annotated using the NCBI GenBank annotation pipeline and RAST genome annotation server (7). A total of 4,446 coding sequences were annotated in various functional categories, including biological processes and metabolism (1,836 genes), cellular components (431 genes), and molecular functions (828 genes). The number of virulence, disease and defense features generated was 91, including adhesion (6.6%), bacteriocins, and ribosomally synthesized antibacterial peptides (13.2%), resistance to antibiotics and toxic compounds (70.3%), and invasion and intracellular resistance (9.9%).

### Accession number(s).

This whole-genome shotgun project has been deposited at DDBJ/ENA/GenBank under the accession no. NAPQ00000000. The version described in this paper is version NAPQ01000000.

## References

[1] Giammanco GM, Pignato S, Mammina C, Grimont F, Grimont PAD, Nastasi A, Giammanco G 2002 Persistent endemicity of Salmonella bongori 48:z35:− in southern Italy: molecular characterization of human, animal, and environmental isolates. J Clin Microbiol 40:3502–3505. doi:10.1128/JCM.40.9.3502-3505.2002. 12202604 PMC130773

[2] Fookes M, Schroeder GN, Langridge GC, Blondel CJ, Mammina C, Connor TR, Seth-Smith H, Vernikos GS, Robinson KS, Sanders M, Petty NK, Kingsley RA, Bäumler AJ, Nuccio SP, Contreras I, Santiviago CA, Maskell D, Barrow P, Humphrey T, Nastasi A, Roberts M, Frankel G, Parkhill J, Dougan G, Thomson NR 2011 Salmonella bongori provides insights into the evolution of the salmonellae. PLoS Pathog 7:e1002191. doi:10.1371/journal.ppat.1002191. 21876672 PMC3158058

[3] Woodward DL, Khakhria R, Johnson WM 1997 Human salmonellosis associated with exotic pets. J Clin Microbiol 35:2786–2790. 9350734 10.1128/jcm.35.11.2786-2790.1997 PMC230062

[4] Bolger AM, Lohse M, Usadel B 2014 Trimmomatic: a flexible trimmer for Illumina sequence data. Bioinformatics 30:2114–2120. doi:10.1093/bioinformatics/btu170. 24695404 PMC4103590

[5] Bankevich A, Nurk S, Antipov D, Gurevich AA, Dvorkin M, Kulikov AS, Lesin VM, Nikolenko SI, Pham S, Prjibelski AD, Pyshkin AV, Sirotkin AV, Vyahhi N, Tesler G, Alekseyev MA, Pevzner PA 2012 SPAdes: a new genome assembly algorithm and its applications to single-cell sequencing. J Comput Biol 19:455–477. doi:10.1089/cmb.2012.0021. 22506599 PMC3342519

[6] Hyatt D, Chen GL, Locascio PF, Land ML, Larimer FW, Hauser LJ 2010 Prodigal: prokaryotic gene recognition and translation initiation site identification. BMC Bioinformatics 11:119. doi:10.1186/1471-2105-11-119. 20211023 PMC2848648

[7] Aziz RK, Bartels D, Best AA, DeJongh M, Disz T, Edwards RA, Formsma K, Gerdes S, Glass EM, Kubal M, Meyer F, Olsen GJ, Olson R, Osterman AL, Overbeek RA, McNeil LK, Paarmann D, Paczian T, Parrello B, Pusch GD, Reich C, Stevens R, Vassieva O, Vonstein V, Wilke A, Zagnitko O 2008 The RAST Server: Rapid Annotations using Subsystems Technology. BMC Genomics 9:75. doi:10.1186/1471-2164-9-75. 18261238 PMC2265698

